# A Longitudinal Dynamic Perspective on Quality in Journalism: Investigating the Long-Term Macro-Level Media Effect of Suicide Reporting on Suicide Rates Across a Century

**DOI:** 10.1177/00936502221150315

**Published:** 2023-03-27

**Authors:** Manina Mestas, Florian Arendt

**Affiliations:** 1 University of Vienna, Austria

**Keywords:** quality in journalism, reporting on suicide, Werther effect, media guidelines, media effects

## Abstract

Quality of journalism is not a stable phenomenon, yet there is limited longitudinal evidence. We provide a content analysis of news reporting over a whole century within a specific thematic context: suicide reporting. Quality is a key dimension in this context as low-quality reporting is associated with imitative suicides (Werther effect). We took a historical perspective: suicide rates increased in many countries during the 19th century, with suicide reporting hypothesized as a contributory factor. Conducting the first longitudinal study of journalism quality that examines an entire century, we manually coded *N* = 14,638 articles. Our analyses indicated a strong nonlinear increase in low-quality reporting. Importantly, a high quantity of low-quality reporting predicted annual increases in suicide rates, a finding which is consistent with the idea of a long-term macro-level media effect. Despite limitations in causal interpretations, the findings support recommendations for high-quality suicide reporting in current media guidelines.

In the 19th century, suicide became a mass phenomenon in many countries (Durkheim, 1897). In Austria, for example, there was a substantial increase in suicides in the two decades between 1860 and 1880 (Kuttelwascher, 1912). Processes of modernization, such as industrialization, urbanization, and secularization, and the decrease in social integration—the weakening of the bond between the individual and society—have been identified as possible risk factors contributing to this increase (Ausenda et al., 1991; Durkheim, 1897; Ortmayr, 1990; Stack, 1993). Importantly, the press has also been noted as a possible contributory factor for increasing suicide numbers (Arendt, 2018; Durkheim, 1897; Masaryk, 1881).

Indeed, among a myriad of factors, contemporary scholars consider the media to be a key contributory factor based on decades of empirical research and theorizing (Mann et al., 2005; Niederkrotenthaler et al., 2014). In fact, suicide reporting has been shown to be a risk factor for imitative suicides, termed the “Werther effect” (Phillips, 1974). Specifically, studies have revealed that a high amount of low-quality suicide reporting has been associated with subsequent increases in suicide rates (Niederkrotenthaler et al., 2020; Stack, 2005). Low-quality reporting, also often termed “sensational suicide reporting” in previous scholarship, has been conceptualized as a reporting style that provides detailed, vivid descriptions of the suicide method, location, and characteristics of the suicidal person, and that gives undue prominence to suicide (see below). It is assumed that especially a high quantity of this kind of suicide reporting increases the risk for imitative effects.

Available evidence on the Werther effect is largely based on either (1) observational (ecological) studies that typically compare suicide rates before and after a target media event, such as the reporting on a celebrity suicide, or (2) individual-level studies that typically use survey designs and assess whether there is a correlation between media-related predictors and suicidality-related target outcomes (see Pirkis & Blood, 2001). Experimental studies on the Werther effect are very rare due to ethical considerations. Although the available studies have substantially contributed to our knowledge, there is clearly a lack of longitudinal assessment. Previous research on the Werther effect (see Niederkrotenthaler et al., 2020; Stack, 2005) has provided evidence showing that a high quantity of low-quality suicide reporting can contribute to an increase in suicide numbers in the short term (i.e., days, weeks, or a few months), yet there is still a lack of knowledge as to whether suicide reporting can also contribute to societal-level changes in suicide numbers in the long term (i.e., years, decades, or even a century). The present study contributes to the literature by investigating the effects of suicide reporting on suicide rates across almost a whole century—a possible *long-term macro-level media effect*. To the best of our knowledge, the long period we examined is unprecedented in previous research.

The present paper also contributes to the contemporary scholarly debate on media guidelines on high-quality, responsible suicide reporting (World Health Organization [WHO], 2017). In fact, we will discuss the implications of our findings in terms of current media guidelines on high-quality news reporting based on empirical data from a whole century. Ultimately, we aim to contribute to contemporary reporting quality due to an enrichment of our understanding of the role of the media in the suicide domain via the historical perspective—the knowledge of the “then” can inform our understanding of the “now.” Thus, we aimed to use “the past to study the present” (Lawrence, 1984, p. 307).

## Quality in Journalism and Suicide Reporting

Quality in journalism is a complex issue, with numerous heated debates on what constitutes “good” journalism. Such debates are not an entirely new phenomenon. For example, Stieler (1695/1969) debated the quality of news reporting in 17th-century newspapers. The quality debate was again stimulated in the 19th century, when the modern mass press gained importance (see Wilke, 2003). More recently, emerging 21st-century technology has been credited with provoking an “existential crisis of journalism” (McNair, 2012), though certain characteristics associated with “quality journalism” may help mitigate these issues.

Much of the previous work on the quality in journalism focuses on journalism’s role in *democracy* (Strömbäck, 2005): Based on distinctive *normative* models of democracy, scholars derive quality criteria, such as acting as a watchdog or fostering public discussion by providing a forum (McQuail, 1992; Voltmer & Rawnsley, 2019). Importantly, proper news standards, that is, the question of what constitutes high-quality journalism, cannot be addressed in isolation from the question of different normative models of democracy, as Strömbäck (2005) argued. Each model of democracy has its own normative implications and leads to different basic demands being placed upon the media. All of these normative models stress the importance of, for example, factually correct information or being consistent with legal requirements (e.g., personality rights). However, what might be considered “good” journalism from the perspective of one normative model of democracy can be judged differently—or might even be considered harmful—from the perspective of an alternative model (Strömbäck, 2005).

The context of *suicide reporting* is different: *Empirical evidence* on media effects, rather than normative theorizing, guides the basic demands placed upon the media. As already noted above, research has revealed that a high amount of low-quality suicide reporting is a risk factor for prompting imitative suicides. Based on this empirical evidence, media guidelines on high-quality responsible suicide reporting have been developed (see Pirkis et al., 2006, for an overview of the guidelines) and disseminated by international (WHO, 2017, 2019) and national organizations (e.g., Wien, 2017). In Austria, where the present study was conducted, suicide coverage is even mentioned in the press codex of the Austrian Press Council as a separate point (Österreichischer Presserat, 2019). In contrast to previous work on the quality in journalism focusing on its role in democracy, quality, as defined in the suicide-prevention domain, is related to the aim of saving lives. The potential risk or, stated differently, the dangerousness of an article triggering harmful effects is central to the conceptualization of quality in the suicide reporting domain. Thus, scholars use the *instrumental argument* to defend the call that the media should adhere to media guidelines by pointing to the robust empirical evidence showing that a high quantity of low-quality reporting can elicit the Werther effect.

Taken together, media guidelines on responsible suicide reporting are *not* primarily based on a distinctive normative model but on the instrumental argument of decreasing the likelihood of detrimental imitative effects—based on the available empirical evidence. Therefore, a broad and deep empirical knowledge base is of utmost importance in the specific domain of suicide reporting. We contribute to this knowledge base by investigating a possible long-term macro-level media effect—unprecedented in previous research.

In an effort to summarize years of empirical research, it is helpful to group media recommendations for high-quality suicide reporting into four categories: method-related information, location-related information, identification-evoking potential, and prominence. First, *method-related information* can facilitate imitative behavior by giving vulnerable individuals a decision-making shortcut and informing them about (and prime) suicide methods, including particularly lethal ones. For example, Chan et al. (2005) interviewed survivors of charcoal-burning suicides and found that nearly all of them had learned about the method via the press, who vividly described the technique and “conveyed an implicit message that charcoal-burning is an easy, painless and effective means of ending one’s life” (p. 69). Furthermore, Yip et al. (2006) observed an increase in suicides by jumping in the immediate aftermath of a famous pop singer’s death by jumping, while Cheng et al. (2007) found an increase in suicides by hanging after a famous TV actor died by hanging. Second, *location-related information* may contribute to the popularity of specific locations (e.g., Golden Gate Bridge). Providing information about location-related facts in articles dealing with suicide, such as the specific location of the suicide or the place where the suicidal individual lived, can lead to the establishment of so-called suicide hotspots (Pirkis et al., 2015). Third, *identification-evoking potential*, that is, personal details such as the deceased’s age, gender, name, or occupation, should be kept to a minimum as including such details can evoke identification processes in the reader. In this regard, research distinguishes between vertical identification (i.e., identification with a celebrity; e.g., Yip et al., 2006) and horizontal identification (i.e., identification by sharing certain characteristics with the deceased; e.g., Schmidtke & Häfner, 1988). Studies have repeatedly found that imitative effects can be especially pronounced under circumstances of high identification (Niederkrotenthaler et al., 2020). Fourth, *prominence* refers to the amount of salience the suicide depiction receives *within* a given article (or newspaper issue). This can manifest, for example, through the article’s appearance on the front page, the use of pictures, or an explicit reference to suicide in the headline in undue fashion. The idea is that high prominence makes it more likely that a given reader will actually read the suicide story (Phillips, 1974).

The available evidence indicates that adherence to media guidelines addressing these content elements can decrease the risk of copycat effects. In a seminal study, Etzersdorfer and Sonneck (1998) investigated the effects of implementing such media guidelines. In the 1980s, the subway system became increasingly popular as a means by which to die by suicide in Vienna. At the same time, news reporting about subway suicides was very sensational (as defined above). When researchers launched a campaign in mid-1987, the quality of news reporting changed, and the number of subway suicides and suicide attempts dropped within a few months by more than 80% from the first to the second half of 1987. According to the researchers, the decrease in suicides was very likely caused by an increase in the quality of journalistic reporting. In fact, several studies since then have suggested that high-quality suicide reporting can contribute to suicide prevention (see Bohanna & Wang, 2012).

## The Present Study: A Longitudinal Dynamic Perspective

In a recent review, Meier (2019) noted an important gap in the research regarding quality in journalism and emphasized that there was limited longitudinal assessment. He argued that future research “could measure even more closely the development of quality by means of longitudinal analysis conducted over several years” (p. 6). A longitudinal perspective is important because quality in news reporting is not a stable phenomenon but can change dynamically over time (Levinsen & Wien, 2011). We argue that this claim holds for an investigation of 19th-century suicide reporting, as we expected an increase in low-quality suicide reporting throughout the 19th century based on three points.

First, there is evidence that sensational reporting, in general, increased during the 19th century, as indicated, for example, by a rise in stories on murder, mayhem, mudslinging, scandals, and disasters, accompanied by the rise of the (low-cost) penny press and the careers of specific editors and reporters being dedicated to this particular journalistic style (Sachsman & Bulla, 2015). Of note, technological innovations such as steam-powered presses, the expansion of the railway network, and the telegraph led to faster news delivery and thus supported the rise of the penny press (see Pensold et al., 2021). Second, a new type of newspaper appeared in Austria in the period before the dramatic increase in suicide rates (Melischek & Seethalter, 2006): local papers. In contrast to the established papers of the liberal press, these papers focused on local news coverage more strongly and thus increasingly included (local) suicide stories using a more vivid narrative style compared to established papers. The latter is problematic when reporting on suicides (see above). Third, there is some preliminary evidence for an increase in low-quality suicide reporting from small-scale content analyses. A content analysis of 160 articles published in Austria provided such evidence of low-quality reporting tending to increase in the second half of the 19th century (Arendt, 2021). Relatedly, Richardson (2015) showed that early Canadian newspapers frequently included details on the suicide, such as vivid descriptions of the exact manner of death. However, the “coverage prior to 1900 was examined (. . .) in a less systematic way” (p. 428) by presenting illustrative reports, rendering generalizations difficult.

Taken together, changes related to trends in general reporting (sensationalism), technological innovations, the emergence of local papers, and preliminary evidence from small-scale content analyses led us to assume that the 19th-century press increasingly relied on *low-quality reporting* in its suicide coverage. Therefore, we formulated a first hypothesis focusing on the four quality concepts outlined above. Note that this hypothesis focuses on the quality of reporting *within a news article*, or stated differently, we expected that we would find an increase in low-quality suicide reporting when examining the “average article” over time.

*Hypothesis 1 (H1).* In the 19th century, there was an increase in low-quality suicide reporting in terms of method (H1.1), location (H1.2), personal details related to identification-evoking potential (H1.3), and prominence (H1.4).

We already noted that modernization processes have been identified as risk factors for increasing suicide rates, with the press recognized as one possible contributory factor. Of course, causal claims in terms of a *long-term macro-level media effect* during the 19th century have to be made with caution (Arendt, 2021): Controlled experiments or a survey of readers are not possible. However, one way of assessing the question of possible press-induced imitative suicides is to collect content-analytic evidence over the whole century and to test its association with suicide numbers. The guiding concept behind this is that current empirical and theoretical knowledge on the Werther effect can identify elements in suicide reporting that are known to contribute to imitative effects. If the content analysis finds evidence that is consistent with contemporary knowledge on imitative effects (i.e., an increase in the quantity of low-quality reporting during a time period of increasing suicide numbers), such findings increase the plausibility of the hypothesis of a long-term Werther effect.

Although some studies provide content-analytic data for suicide reporting in the 19th and beginning of the 20th centuries (Arendt, 2018; Richardson, 2015; Wasserman et al., 1994), little is known about the nature of the interplay between suicide rates and the press back then. Previous work provides preliminary evidence for a covariation between the quantity of suicide reporting and suicide rates (Arendt, 2018). In fact, the evidence indicated that the press’s quantity of suicide reporting tended to mirror the peaks and troughs in suicide rates over time: The higher the number of suicide articles that appeared in the press in a given year, the higher the suicide rate was. Unfortunately, the quality of the reporting was not assessed.

While it is extremely difficult to establish causality for press-induced increases in suicide rates over a hundred years later, one would expect a covariation pattern: If the press contributed to macro-level suicide rates, there should be a covariation between a *high quantity of low-quality reporting* and suicide rates. The basic idea is as follows: If we find a statistical association consistent with the Werther effect *and* we combine this finding with the available empirical knowledge from contemporary scholarship, it is plausible to assume that the press might have contributed to suicide numbers. Therefore, we hypothesized that there would be a statistical association between a high amount of low-quality reporting and increasing suicide rates. This hypothesis focuses on the quantity of low-quality reporting given that the quality *and* quantity dimensions are relevant for an expected long-term macro-level media effect on imitative suicides (Niederkrotenthaler et al., 2020; Phillips, 1974; Stack, 2005). What counts in this regard is whether the quantity of low-quality reporting in the entire news environment *of the press* changed and whether these changes were related to changes in societal-level suicide rates. Thus, we formulated a second hypothesis related to a possible long-term macro-level media effect:

*Hypothesis 2 (H2).* There was covariation between the quantity of low-quality reporting and suicide rates, with a higher quantity of low-quality reporting predicting higher suicide rates.

We want to emphasize that both hypotheses follow a different analytical logic: Whereas H1 focuses on *low-quality reporting* in a given article per se using the target concepts of the method, location, identification-evoking potential, and prominence (i.e., how responsible is a suicide depiction *within a given article* and does the quality of reporting of the “average article” change over time?), H2 focuses on a possible long-term macro-level media effect. The latter thus switches the logic of the analysis and relies on the *quantity of low-quality reporting* by the press, as this is the relevant factor for the study of a *macro-level* media effect. Theoretically speaking, it is the quantity of low-quality reporting that indicates the strength of the Werther effect-evoking potential of the suicide coverage in the entire message system of *the press*.

## Method

To assess the quality of suicide reporting, we conducted a large-scale content analysis of suicide reporting between 1819 (first suicide statistics available) and 1899 in the geographic region of present-day Austria. The first aim was to provide a thorough description of suicide reporting in the 19th-century press, focusing on the quality of reporting in the “average article” over time (see H1). We measured the quality of suicide reporting by relying on target concepts emphasized in current media guidelines. We also compared the historical data to a reference time (i.e., the present) to determine similarities and differences in the quality of journalism between then and now. Thus, we also coded the same indicators for current-day suicide reporting in three consecutive years (i.e., 2018–2020). The second aim was to investigate a possible long-term macro-level media effect of suicide reporting on suicide rates (see H2).

### Sample

Relying on the Austrian National Library’s online archive ANNO, we selected 20 German-speaking newspapers with high circulation in the corresponding period and relevant region. We included newspapers from different territories of the monarchy and of different types, including liberal, political, and official press, local papers, and papers of the emerging mass press (see Melischek & Seethalter, 2006, for an overview of the Austrian press at the time). A full list of all the newspapers can be found in the Online Supplemental Material Document (OSM Table 1). The coders used the online ANNO archive to search for articles using the keyword “Selbstmord” [suicide] to code *n* = 30 randomly selected suicide articles per newspaper-year combination. It is worth noting that most newspapers were not published during the entire observation period, and some newspaper-year combinations did not produce 30 search results (especially during the first half of the century, see below). Our final 19th-century sample comprised *N* = 14,638 manually coded suicide articles.

To compare the historical data with today’s suicide reporting in the Austrian press, we analyzed articles in the 10 Austrian newspapers with the widest circulation available in the Austrian Press Agency (APA) database using multiple (modern-day) keywords that denote “suicide” (i.e., “Suizid,” “Freitod,” “Selbsttötung”), as well as the word used in the 19th century (i.e., “Selbstmord”). We included three consecutive years (2018–2020) and drew a sample of *n* = 10 randomly selected suicide articles per newspaper per year, resulting in *N* = 300 articles as our present-day sample.

### Procedure

To ensure high measurement reliability, four rounds of coder training with reliability tests with *N* = 17 coders were conducted until the Krippendorff’s alphas of all the variables indicated high intercoder reliability. The data collection for the main sample was conducted over 2 months. Each coder was randomly assigned to code a subset of the sample, which was randomly distributed over the whole century and the various newspapers. To ensure high data quality and to assess intercoder reliability during the actual coding process, the same three newspaper–year combinations were given to all coders without the coders knowing that all the other coders also coded this news content.

### Low-Quality Reporting

To test H1 (whether the quality of reporting within a given article changed over time), we measured the following four quality-related variables *for each article*: method, location, identification-evoking potential, and prominence. For each quality variable, we asked the coders to assess the presence of several content elements. Each quality variable was operationalized as the sum of its content elements. The higher the score, the lower the quality of reporting. Table 1 presents all of the content elements for the four variables and provides descriptive statistics, including intercoder reliability estimates from the actual coding process.

**Table 1.** Four Variables to Measure the Quality of Suicide Reporting.

Variable	Operational definition	Statistics		
		*M*	*SD*	α
Method	This variable indicates whether and to what extent the article provides method-related information. Coders assessed a total of four content elements (0 = not present, 1 = present), that is, whether (1) the article describes the suicide method (e.g., poisoning), whether (2) the article includes details of the suicide method (e.g., died by suicide after having ingested cyanide pills), whether (3) the picture depicts the suicide method, and whether (4) the suicide method is mentioned in the headline. The measure represents the sum of the content elements for each article (possible range = 0–4).	1.50	0.77	.88
Location	This variable indicates whether and to what extent the article provides location-related information. Coders assessed a total of three content elements (0 = not present, 1 = present), that is, whether (1) the article mentions the location of the suicide, whether (2) the article mentions the place where the suicidal individual lived, and whether (3) the article includes the specific address of the suicidal individual. The location of the suicide, the place of residence, and the specific address all provide location-related information. The measure represents the sum of the content elements for each article (possible range = 0–3).	1.46	0.83	.66
Identification	This variable indicates whether and to what extent the article reports on personal details of the suicidal individual. The more personal details that are mentioned, the higher the identification-evoking potential. Coders assessed a total of six content elements (0 = not present, 1 = present), that is, whether the article mentions the suicidal individual’s age, gender, occupation, societal status (i.e., aristocracy, celebrity), first name, and last name. The measure represents the sum of the content elements for each article (possible range = 0–6).	3.22	1.43	.82
Prominence	This variable indicates whether and to what extent the article gives undue prominence to suicide. Coders assessed a total of three content elements (0 = not present, 1 = present), that is, whether (1) the article appears on the front page of the newspaper, whether (2) the article mentions the suicide in the headline, and whether (3) the suicide article is accompanied by a picture. The measure represents the sum of the content elements for each article (possible range = 0–3). The higher the score, the higher the presumed likelihood that the suicide article attracted a given reader’s attention.	0.66	0.48	.90

*Note.* The higher each score, the lower the quality of reporting is (and the higher the presumed Werther effect-evoking potential). *Ms* = Means and *SDs* = standard deviations were calculated based on all 19th-century articles (*N* = 14,638). We used Krippendorff’s α to assess intercoder reliability for the actual coding process.

### Quantity of Low-Quality Reporting

To test H2 (i.e., to analyze whether a high quantity of low-quality reporting was related to suicide rates), we had to construct a variable that combined the quality *and* quantity variables. (Note that the theory outlined above predicts that a high quantity of low-quality reporting is related to increases in the number of suicides.) Therefore, we also had to assess the quantity of suicide reporting. We did this for *each year* within the observation period given that we used a macro-level long-term perspective relying on annual data. We used two quantity-related indicators, termed *sum* (i.e., sum of suicide articles per year across all newspapers) and *mean* (i.e., mean annual number of days across all available newspapers on which a reader was confronted with a suicide article), that could be assessed in the historical newspaper archive ANNO with simple keyword searches. The higher these two measures were, the more frequently the press reported on suicide in a given year.

To measure the quantity of low-quality reporting, we used the six variables described above (i.e., the four quality measures and two quantity measures) and ran a factor analysis, resulting in a one-factor solution (i.e., only one factor had an eigenvalue greater than 1.0). This factor, *eigenvalue* = 4.15, explained 69.17% of the variance, and all six variables showed high factor loadings. We used the factor scores for further analysis. The higher this score was, the higher the quantity of low-quality reporting was in a given year.

### Suicide Rate

We used suicide rates from Ortmayr (1990, p. 221), which are based on Kuttelwascher (1912, p. 285), representing the annual number of suicides per 100,000 people for each year of the observation period.

### Controls

As a supplement to the bivariate time-series analysis to test H2 (i.e., covariation between a high quantity of low-quality reporting and suicide rates), we used additional variables to examine other societal-level change processes. We used these additional variables as covariates to check for a possible spurious relationship. The aim was to determine if an observed statistical association between the press and suicide rates would hold when controlling for these third variables. The selection of covariates was largely determined by the availability of 19th-century Austrian time-series data—we needed the annual time-series data from 1819 to 1899 to serve as a proxy for societal-level change processes. We used indices for the (1) *economy* (i.e., Consumer Price Index: a central indicator for the economy; Mühlpeck et al., 1994), (2) *politics* (i.e., Democratization Index: an index that combines two basic dimensions of democracy—competition and participation—and provides information about the level of democratization; Vanhanen, 2000), and *social and cultural factors*. Regarding the latter two, we relied on the Historical Varieties of Democracy (Historical V-Dem) Project (V-Dem, 2022), which uses expert coding to create annual time-series data: (3) freedom of academic and cultural expression (Is there academic freedom and freedom of cultural expression?), (4) freedom of religion (Is there freedom of religion?), (5) the Women’s Civil Liberties Index (Do women have the ability to make meaningful decisions in key areas of their lives?), and (6) the Rule of Law Index (To what extent are laws transparently, independently, predictably, impartially, and equally enforced, and to what extent do the actions of government officials comply with the law?).

### Statistical Analysis

*To test H1*, we investigated the bivariate functional relationships between time (*X*) and the quality concepts (*Y*). In the first step, we assessed the means, standard deviations, and number of available articles for our *four target quality variables* (i.e., method, location, identification-evoking potential, and prominence) *for each year* and used these annual scores as input values for further analysis. Thus, we were interested in the quality of reporting in the “average article” within a given year. Given that we expected nonlinear *s*-curve-type trends, based on previous research (Arendt, 2018), we used nonlinear regression analysis to fit sigmoidal functions, using PRISM (GraphPad Software, Inc.). We fitted four-parameter logistic models, *Y* = *bottom* + (*top*−*bottom*)/[1 + 10^((*IP*−*X*) × *slope*)], where *Y* is the outcome (quality concept), and *X* is time. The *bottom* estimates the bottom plateau, and the *top* estimates the top plateau. *Top-bottom* assesses the range (i.e., variation) in the quality of journalistic news reporting observed across the whole century. *IP* estimates the *s*-curve’s inflection point, located halfway between the bottom and top. Thus, *IP* estimates the year with the strongest annual increase in low-quality reporting. The *slope*, also called the slope factor or Hill coefficient, estimates the steepness of the curve. If the slope is positive, the curve increases as *X* increases, and higher positive slope values indicate that the curve is steeper.

Comparing the historical data to today’s suicide reporting in the Austrian press, we calculated the means across all 3 years of recent reporting (i.e., 2018–2020), including their confidence intervals (95%), based on bootstrapping techniques.

To assess covariation and thus a possible long-term macro-level media effect (H2), we relied on (1) an autoregressive integrated moving average (ARIMA) model to test whether a *high quantity of low-quality* reporting was related to suicide rates. An ARIMA model can be viewed as a special type of regression model in which the independent variables are lags of the dependent variable (autoregressive terms) and/or lags of the errors (moving average terms; see Jebb et al., 2015). Importantly, an ARIMA model that accounts for autocorrelation can be extended to incorporate information provided by an exogenous conceptual variable, such as quantity of low-quality reporting. The latter variable was added as a regressor. Such models can be termed dynamic regression models—that is, regressions with ARIMA errors. Furthermore, we (2) used a Granger-causality test (Granger, 1969) to assess whether there was evidence for a specific causal order between the quantity of low-quality reporting and suicide rates. We provide more details below.

Given the fact that there are many other factors that may have contributed to changes in the suicide rate, we re-ran the ARIMA model and the Granger-causality test by including the six additional control variables outlined above as covariates. This additional analysis was performed to test whether the effects of the press on suicide rates remained when controlling for the third variables.

### Ethics Statement

The project was approved by the Institutional Review Board of the Department of Communication, University of Vienna (Number ID: 20210414_026, dated May 3, 2021). To ensure coder safety, the coders were screened for heightened levels of hopelessness (Beck & Steer, 1988) before, during, and after coding. During coder training, the fact that suicide articles can elicit negative effects, such as emotional discomfort, was discussed. We urged coders to be aware of this phenomenon, reminded them of it throughout the coding process via supervision, and used regular “mental health check-ins” by the project staff.

## Results

### Quality of Reporting Within Suicide Articles Over Time

H1 predicted an increase in the following four target quality concepts over time: method (H1.1), location (H1.2), identification-evoking potential (H1.3), and prominence (H1.4). Stated differently, we predicted that the “average article” got worse (from a suicide-prevention standpoint) in terms of these four target quality concepts during the 19th century. For each of the four target quality concepts, we calculated the mean and standard deviation for each year across all available articles in that given year (see OSM Table 3 in the Online Supplemental Material Document). Next, we used nonlinear regression analysis to test this hypothesis, employing the mean, standard deviation, and number of available articles each year to fit a sigmoidal function. It is worth noting that the number of available articles was rather small in many years in the first half of the century (i.e., one-digit numbers—some years only one article—compared to three-digit numbers in the second half of the century). However, curve fitting with nonlinear regression weights the influence of individual data points (i.e., years), adjusting for the number of available articles. For example, a year for which 50 articles were available weights more in the curve-fitting procedure compared to a year in which only five articles were available. Therefore, we used all the years for analysis, even when the number of suicide articles in the newspaper archive was small. Note that this analysis related to H1 did *not* include the quantity of reporting but focused on whether the quality of reporting within the “average article” worsened over the years.

**Figure 1.**
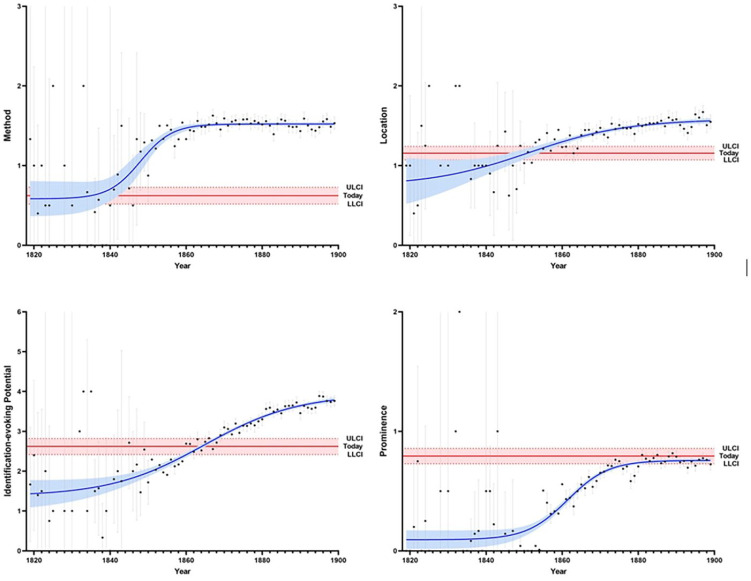
Dynamic change of four quality concepts over the century: method, location, identification-evoking potential, and prominence. *Note*. A higher score indicates a lower quality of journalistic news reporting. Each dot represents the mean for the corresponding quality variable for each year, with the gray vertical lines indicating the confidence intervals of individual annual data points (95%). The blue curves are based on nonlinear regression analyses and represent the fitted sigmoidal functions (including their confidence bands, 95%). The red horizontal line shows today’s level. LLCI = lower limit confidence interval (95%); ULCI = upper limit confidence interval (95%).

Figure 1 shows that the annual scores for each of the four quality concepts increased throughout the century, as predicted. Note that each data point represents the mean for the respective target quality concept in a given year, including its confidence interval (95%). The fitted sigmoidal function is based on nonlinear regression analysis (curve fitting) that weights the influence of individual data points based on the number of available articles in a given year. This curve is thus adjusted for the number of available articles in a given year. The calculation of the confidence bands (95%) for the fitted sigmoidal curve—the estimated bands that enclose the area that we can be 95% certain contains the true curve—is also based on nonlinear regression analysis.

On the top left of the figure, we present the findings related to the *method*: The curve started off at today’s rather low levels, rose between approximately 1840 and 1860, and plateaued at a high level, with very little variation for the remainder of the century. Contrarily, the curve for the *location*, found on the top right of Figure 1, was less steep. It started out below today’s levels, rose slowly to surpass today’s values around mid-century, and continued to rise until flattening in the last quarter of the century. The course of the means for the *identification-evoking potential*, found on the bottom left of Figure 1, also started out rather low, exceeded today’s value around 1860, and continued to rise over the remaining period. Finally, the fitted *s*-shaped curve for *prominence*, found on the bottom right of Figure 1, started at a very low level (almost zero) and increased between around 1850 and 1880, when it plateaued at a level similar to present-day suicide reporting.

Taken together, the evidence shows an increase in the method, location, identification-evoking potential, and prominence in 19th-century suicide reporting. Thus, all four quality indicators worsened substantially within a few decades. The data indicate an increase in low-quality reporting, especially during the second half of the century. Reporting quality was clearly not stable over time but rather indicates dynamic change. Hence, the analysis supports H1.

### Toward A Long-Term Macro-Level Media Effect

We now switch the logic of the analysis, as we focus on a possible macro-level media effect of a *high quantity of low-quality* suicide reporting on suicide rates. In fact, H2 predicted a covariation between the quantity of low-quality reporting and suicide rates, with a higher quantity of low-quality reporting related to higher suicide rates. The following analysis related to H2 relied on a variable that combines quality *and* quantity concepts (i.e., quantity of low-quality suicide reporting) as the theory outlined above predicts that a high quantity of low-quality reporting is related to the number of suicides.

To test this hypothesis, we relied on a two-step approach. First, we used a nonlinear regression and compared the two functions of both time series (i.e., quantity of low-quality reporting and suicide rates). This intuitive first step allowed us to investigate whether the increase in one time series descriptively preceded the other. Second, we conducted formal tests of statistical association by relying on a time-series analysis (i.e., ARIMA modeling and a Granger-causality test).

### First Step: Comparing Both Curves

**Figure 2.**
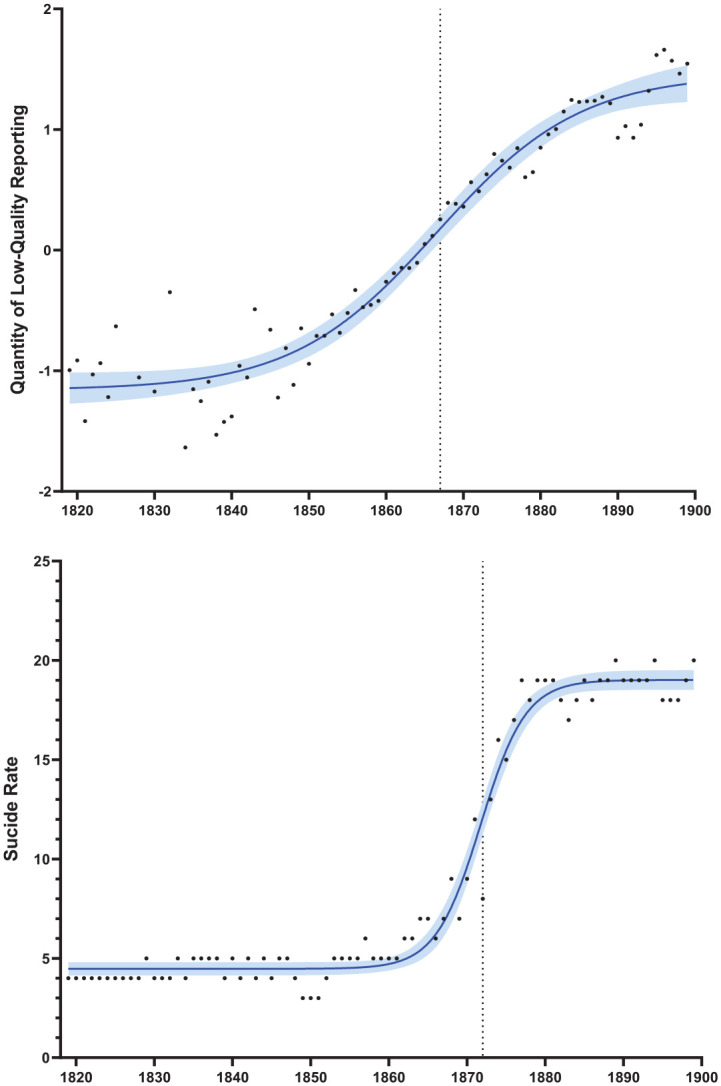
Quantity of low-quality reporting and the suicide rate across the 19th century. *Note*. Curves represent fitted sigmoidal functions based on nonlinear regression analyses, including their confidence bands (95%). The vertical dotted lines represent the *s*-curve’s inflection points—that is, the year pointing to halfway between the bottom and top of the *s*-curve.

Figure 2 depicts the quantity of low-quality reporting and suicide rates. We obtained a *s*-curve-type increase when examining the quantity of low-quality reporting, *bottom* = −1.16 [−1.30, −1.04], *top* = 1.46 [1.27, 1.73], *IP* = 1867 [1864, 1869], *slope* = 0.05 [0.04, 0.06], *df* = 71, *R*² = .95. (We report 95% confidence intervals in squared brackets.) Six years had missing values (for 5 years, there was an absence of any coding material, and 1 year was identified as an outlier), explaining the reduced number of degrees of freedom. The curve indicates that the quantity of low-quality reporting started at a low level, showed the sharpest increase in 1867 (inflection point), and then rose to high values.

We obtained a similar *s*-curve-type increase when examining suicide rates. The graph shows a strong increase in suicides between approximately 1860 and 1880. As data appeared in an *s*-curve form, we fitted a sigmoidal function, *bottom* = 4.47 [4.18, 4.77], *top* = 19.02 [18.58, 19.46], *IP* = 1872 [1871, 1872], *slope* = 0.15 [0.12, 0.19], *df* = 77, *R*² = .98. This indicates that suicide rates started at a low level (i.e., about 4 suicides per 100,000 people), showed the strongest annual increase in 1872 (inflection point), and subsequently reached a high plateau value (about 19 suicides per 100,000 people).

Figure 2 visualizes the curves’ inflection points (i.e., 1867 and 1872) with vertical dashed lines. It is worth noting that the increase in the quantity of low-quality reporting started earlier, as indicated by an earlier inflection point (the confidence intervals of both inflection points, reported above, do not overlap). There was also evidence that the curve was shallower (again, note that the confidence intervals of both slopes did not overlap; suicide rates showed a steeper increase). This analysis indicates that while both suicide rates and the quantity of low-quality reporting increased, the *increase in the quantity of low-quality reporting preceded the increase in suicides*.

### Second Step: Formal Tests Using Time-Series Analysis

#### ARIMA Modeling

In the analysis described above, we used a nonlinear regression to *describe* the functional relationship between time and the outcomes (i.e., the trend over time). To test specifically whether covariation existed between suicide rates and the quantity of low-quality reporting (i.e., whether the quantity of low-quality reporting helps *predict* suicide rates), we relied on ARIMA modeling to account for the autocorrelation. The latter is important for time-series data (Schaffer et al., 2021). We used suicide rates as the outcome and the quantity of low-quality reporting as the predictor. Given that the annual estimates for the quantity of low-quality reporting appeared to be very imprecise in the first half of the 19th century due to the low number of codable observations (see Figure 2) and six missing values (see above), we used the simulated (i.e., predicted) values of the fitted *s*-curve as the predictor variable in ARIMA modeling. We also used first differencing for this regressor. As recommended by Hyndman and Athanasopoulos (2014), we ran a KPSS test (Kwiatkowski et al., 1992), which indicated that first differencing was sufficient to provide a stationary time series. Therefore, we prespecified the *d* term in the ARIMA model (*d* = 1). Next, we used the *auto.arima* function from the *forecast* package in the statistical software *R* to automatically select the most appropriate values for the remaining terms, that is, *p*, *q*, *P*, *D*, and *Q*, which define an ARIMA model (see Schaffer et al., 2021). The function conducts a search of possible models and reports the best ARIMA model according to the AIC, AICc, or BIC values. This procedure identified an ARIMA (3,1,0) model, supporting our decision. This ARIMA model accounted for the autocorrelation in the time series as indicated by the Ljung-Box Q statistic on residual autocorrelation that was not significant (*p* = .97).

Most importantly, the analysis indicated a significant effect for the quantity of low-quality reporting, *B* = 7.43, *SE* = 2.30, *z* = 3.22, *p* = .001. This finding indicated a substantial positive covariation between the quantity of low-quality reporting and suicide rates—that is, the higher the annual increase in the quantity of low-quality reporting, the higher the annual increase in suicide rates.

We re-ran this analysis by additionally including control variables related to the economy, political system, and social/cultural factors. This analysis also indicated a significant effect for the quantity of low-quality reporting, *B* = 7.40, *SE* = 2.77, *z* = 2.68, *p* = .007, indicating that the relationship between the press and suicide rates remained when controlling for third variables related to other societal-level change processes. The full model can be found in OSM Table 2 in the Online Supplemental Material Document. This analysis supports H2.

#### Granger-Causality Test

The ARIMA modeling reported above identified a *covariation* pattern. We conducted additional analysis to formally test whether there is evidence for a specific *causal order* between suicide rates and the quantity of low-quality reporting. In fact, we tested for Granger causality (Granger, 1969). The concept of Granger causality assumes that the time series of one given variable *x* (in our case the quantity of low-quality reporting) “Granger-causes” the time series of another variable *y* (in our case suicide rates) if the lagged values of *x* (*t*–1, *t–*2, etc.) provide statistically significant information about the future values of *y* (*t*) when simultaneously controlling for the lagged values of *y* (*t*–1, *t*–2, etc.). The idea is that the cause must precede the consequence.

In a first step, we used the *VARselect* function of R’s *vars* package to identify the optimal lag structure. This function returns information criteria for sequentially increasing the lag order. Three lags were identified. Note, this is consistent with the ARIMA model reported above, which also used three autoregressive terms. In a second step, we ran the Granger-causality test, using the *grangertest* function of R’s *lmtest* package. The analysis relied on a Wald test that compared the unrestricted model (i.e., the suicide rate is predicted by the three lags of suicides rates and quantity of low-quality reporting) and the restricted model (i.e., the suicides rate is only predicted by three lags of suicide rates). Note that we also used first order differencing, as in the ARIMA model reported above. We found evidence for a bidirectional influence: Quantity of low-quality reporting Granger-caused suicide rates, Wald’s *F* = 3.23, *p* = .027. In addition, suicide rates also Granger-caused quantity of low-quality reporting, Wald’s *F* = 3.82, *p* = .014.

We re-ran this analysis by using a vector-autoregressive model using the *VAR* function of R’s *vars* package. This multivariate test relies on an *F*-type Granger-causality test. We defined the six control variables as additional exogenous variables. We also used first-order differencing for the controls. This analysis also indicated that the quantity of low-quality reporting Granger-caused suicide rates, *F*(3, 128) = 2.75, *p* = .045. In addition, suicide rates also Granger-caused the quantity of low-quality reporting, *F*(3, 128) = 2.70, *p* = .049. Therefore, the findings are very similar when controlling for the third variables related to the economy, politics, and social and cultural factors. Taken together, the findings support H2.

## Discussion

Previous scholarship has acknowledged that quality in journalistic news reporting is not necessarily a stable phenomenon but can dynamically change over time. However, a recent review of the research on quality in journalistic news reporting (Meier, 2019) highlighted the lack of longitudinal assessment. This claim holds for the topical area of the present study: suicide reporting. Quality is a key dimension in this context, as a high amount of low-quality suicide reporting has been associated with imitative suicides (Werther effect). Low-quality reporting on suicide is thus a reporting style that is potentially dangerous. To fill the research gap related to long-term assessment, we utilized a longitudinal dynamic perspective and provided a content analysis of the quality of news reporting *over nearly a century*. We manually coded almost 15,000 newspaper texts, and our analyses indicated a strong nonlinear annual increase in low-quality reporting during the 19th century. All four quality indicators became substantially worse within a few decades.

### Dynamic Change Over Time

The data clearly indicate a dynamic change in the quality of reporting within the “average article,” underlining the fact that the quality of reporting was not stable over time. Notably, the range in the quality of reporting was substantial across all four indicators when comparing the lowest and highest scores over the whole century. Note that the study’s longitudinal perspective highlighted the dynamic nature of quality in journalistic news reporting; if we had only looked at the end of the 19th century (e.g., the last 10 years of the century), for example, the quality of reporting would have appeared relatively stable. However, examining an extended period enabled us to reveal fundamental differences in the quality of reporting.

We want to emphasize that the observed change was *dynamic*—that is, the strength of the annual change was non-constant over time (see the sigmoidal functional relationships visualized in Figure 1). Thus, even if one had looked at, for example, a decade within a period with an increase of the target outcomes (e.g., the 1860s for identification-evoking potential, see Figure 1) and analysis had revealed a linear trend within this period, it would have been incorrect to extrapolate this linear trend as the annual increase was *not* constant and instead dynamically changed over time. Only the longitudinal perspective of using data from nearly a whole century revealed this dynamic change pattern.

### Was There a Long-Term Macro-Level Media Effect?

In the 19th century, suicide became a mass phenomenon in many countries, including the geographic region of the present state of Austria, and scholars have questioned whether the press contributed to increasing suicide numbers (see above). Unfortunately, there was limited empirical evidence available in this regard. Therefore, the present study aimed at investigating a possible long-term macro-level media effect.

On a most basic level, the analysis indicated a statistical association between a high quantity of low-quality reporting and suicide rates: A formal test of covariation using ARIMA modeling techniques revealed a statistically significant association between the quantity of low-quality reporting and suicide rates, even after controlling for autocorrelation and trends in the time series. In addition, we found that both the high quantity of low-quality reporting and suicide rates showed an increase over time, especially in the second half of the century. Importantly, the increase in the high quantity of low-quality reporting seemed to *precede* the increase in suicide rates when comparing both time series. This finding makes chronological sense from a media effects perspective, focusing on the fact that the increase in the media variable “occurred” before the increase in suicide rates. As the cause must precede the consequence, this pattern is consistent with a causal long-term contribution of a high quantity of low-quality reporting to increasing suicide rates. This finding allows us to evaluate *temporality*—an important criterion to judge causality (see Hill, 1971, Pirkis & Blood, 2001). Note that the Granger-causality test provided evidence for a bidirectional causal order between the quantity of low-quality reporting and suicide rates, indicating that the quantity of low-quality reporting stimulated suicide rates, and that a higher number of suicides, in turn, stimulated the quantity of low-quality reporting. It is important to emphasize that the statistical effects obtained remained significant when controlling for other societal-level change processes.

Our (historical) data are undoubtedly limited in terms of causal interpretations. For example, we do not know whether individuals who died by suicide actually read suicide reports. Although we are very cautious when interpreting causal effects, we argue that combining our longitudinal macro-level approach with an approach which “zooms in” can help to increase our confidence in causal interpretations: One case study (Arendt & Mestas, 2021) investigated possible imitation effects for the historical suicide case of pre-World War I spy Alfred Redl, a colonel in the Imperial and Royal General Staff and Deputy Director of Military Intelligence for the Habsburg Empire. This “zoomed in” case study indicated that substantially more people died by suicide in the weeks after his death relative to numerous control periods. Of note, the number of (low-quality) news reports on Colonel Redl predicted the number of suicides per day—a pattern consistent with the Werther effect. Used in combination, we argue, such case studies on rather short-term effects *and* our longitudinal macro-level approach add to our understanding of possible media effects back then. In fact, we think that the available evidence makes the assumption that sensational suicide reporting back then at least partially contributed to suicide numbers plausible, among an intertwined set of other factors. However, we acknowledge that we cannot prove a causal effect with our historical data. More research is definitely needed. We offer post hoc theorizing as a starting point for future research.

When theorizing on the substantial effects of *highly salient media events* such as the suicide of Colonel Redl, we conceptualize the media impact as a rather short-term *drench effect* (Greenberg, 1988). Consistent with Greenberg’s figurative language, such media events may be seen as extraordinary “thunderstorms,” which are potent in terms of eliciting substantial short-term increases in suicide numbers. However, such media events typically do *not* elicit a measurable *long-term* lasting influence on *macro-level* suicide rates. Thus, we conceptualize a *long-term macro-level media effect as a cumulative effect* arising from a high number of media events—including both (1) highly salient “drench events,” such as the suicide of Colonel Redl, *but also* (2) (certainly more numerous) “drip events,” such as brief stories about the suicide of unknown “ordinary” people. Using figurative language, the latter may be seen as episodes of “light drizzle” and may contribute to a gradual, cumulative, *drip-drip-drip effect* (Greenberg, 1988). Drench *and* drip effects (i.e., relatively rare “thunderstorms” and highly frequent episodes of “light drizzle”) may cumulatively sum up to a measurable long-term macro-level media effect. The question of how exactly this happens (i.e., the role of and the interplay between drench and drip media events) is unanswered and is a valuable starting point for future research. Note that this question is also of utmost relevance today.

### Implications for Current Media Guidelines

As already noted above, media guidelines on high-quality responsible suicide reporting are based on the available empirical evidence on media effects. Therefore, a broad and deep empirical knowledge base has been emphasized to be of utmost importance. The present research contributes to this knowledge base by investigating a possible long-term macro-level media effect and thus also has implications for current media guidelines: We interpret our findings against the backdrop of recommendations on high-quality responsible suicide reporting in the current-day media guidelines. While acknowledging limitations in terms of causal interpretations, the evidence in our study is consistent with the claim that nonadherence to quality standards can contribute to an increase in suicides. We thus provide supplementary empirical evidence for previous work on media guidelines (Bohanna & Wang, 2012; Etzersdorfer & Sonneck, 1998; Pirkis et al., 2006). “Using the past to study the present” (Lawrence, 1984), our aim was to draw conclusions from the 19th century to apply to the 21st century.

Note that our data showed that today’s suicide reporting still leaves room for improvement. In fact, the analysis indicated that today’s reporting is of a lower standard when compared to the first half of the 19th century on the quality concepts of location and identification-evoking potential (see Figure 1). Studies based on interviews with journalists also indicate this potential for improvement (Beam et al., 2018; Markiewitz et al., 2019), with findings revealing journalists’ competing motivations when considering whether (and if, then how) to report on a given suicide. While many journalists seem to be aware of the possible detrimental effects of a specific kind of suicide reporting, the urge to report comprehensively and clearly, as well as to meet other quality standards, often opposes current media guidelines on responsible suicide reporting, as does the captivating power of sensationalism, its marketable qualities, and other competing interests (Brosius & Ziegler, 2001).

### Future Research on the Quality of Journalism

To the best of our knowledge, a longitudinal study using data over a whole century is unprecedented in the research on quality in journalism. Although resource intensive, such a longitudinal content analysis allows for unique insights. While this project focused on suicide reporting, similar longitudinal research can be helpful in many topical areas of research on the quality in journalism, such as (1) political news coverage with possible consequences for indicators related to a well-functioning democracy, (2) stereotypical news with possible consequences for indicators related to discriminatory behaviors and an open and humane society, (3) polarized news coverage and possible consequences for affective polarization processes, and (4) other topical areas with possible consequences for imitative behaviors. An important example of the latter may be mass shootings—refraining from providing details may help prevent imitative behaviors in this context (Meindl & Ivy, 2017). We think that more “quality in journalism” studies using a longitudinal dynamic perspective would substantially contribute to scientific progress.

### Limitations

This study has several limitations. First, our content analysis relied on the availability of newspapers in the ANNO archive. We used the search term “Selbstmord” [suicide], which means that we may have missed reports that classified suicide as “self-inflicted death” or used other words to describe the suicide (such as the method) without using the term itself. Second, our search relied on ANNO’s text-identification software, which may misidentify some words as the newspapers were written in *Frakturschrift*, an ancient German typeface. Third, despite extensive pretesting and coder training—including a total of four intercoder reliability test rounds *before* the actual coding process was undertaken—we obtained a rather low Krippendorff’s alpha value of .66 for our location variable *during* the actual coding process (see Table 1). While suboptimal, we decided to use the location variable in a planned analysis as the alpha approaches .70—a value which we set as the threshold during the pretesting and coder training process. Fourth, the number of suicides may have been underreported due to personal or religious reasons or due to a difference in suicide statistics in different territories in the period under consideration (Kuttelwascher, 1912). Fifth, current recommendations found in media guidelines (e.g., by the WHO) call for the omission of Werther effect-facilitating elements such as the four concepts assessed in our study. Instead, possibly helpful content elements, such as providing a role model story (i.e., a report about a person who successfully overcame a suicidal crisis) or presenting contact information on where to find professional help (e.g., a crisis intervention center or telephone counseling service), should be included in high-quality suicide reports, according to media guidelines. In fact, the latter helpful content elements may contribute to a suicide-protective effect, termed the “Papageno effect” (see Niederkrotenthaler et al., 2010, 2022). The Papageno effect was not the focus of the present paper. A systematic longitudinal assessment of Papageno effect-related reporting back then is left for a future study.

## Conclusion

Despite its limitations, this study presents evidence that quality in journalistic reporting is not a stable phenomenon but can reflect dynamic change over time. We also provide supporting evidence for the idea of a long-term macro-level media effect. The utilization of a longitudinal assessment and the study of a possible long-term macro-level media effect is, to the best of our knowledge, the first of its kind.

## Supplemental Material

sj-docx-1-crx-10.1177_00936502221150315 – Supplemental material for A Longitudinal Dynamic Perspective on Quality in Journalism: Investigating the Long-Term Macro-Level Media Effect of Suicide Reporting on Suicide Rates Across a Century Supplemental material, sj-docx-1-crx-10.1177_00936502221150315 for A Longitudinal Dynamic Perspective on Quality in Journalism: Investigating the Long-Term Macro-Level Media Effect of Suicide Reporting on Suicide Rates Across a Century by Manina Mestas and Florian Arendt in Communication Research
