# Supplementary material for: A Longitudinal Dynamic Perspective on Quality in Journalism: Investigating the Long-Term Macro-Level Media Effect of Suicide Reporting on Suicide Rates Across a Century
Source: Communic Res. 2023 Mar 27;51(8):927–51. doi: 10.1177/00936502221150315 (PMC11537977; doi:10.1177/00936502221150315)
Supplement: sj-docx-1-crx-10.1177_00936502221150315 – Supplemental material for A Longitudinal Dynamic Perspective on Quality in Journalism: Investigating the Long-Term Macro-Level Media Effect of Suicide Reporting on Suicide Rates Across a Century [file sj-docx-1-crx-10.1177_00936502221150315.docx]

**ONLINE SUPPLEMENTAL MATERIAL DOCUMENT**

**OSM Table 1**

*List of Newspapers*

| Newspaper type | Name | Years included |
| --- | --- | --- |
| Liberal, political, official press | Die Presse | 1848­–1896 |
|  | Neue Freie Presse | 1864–1899 |
|  | Das Vaterland | 1860–1899 |
|  | Arbeiter-Zeitung | 1890–1899 |
|  | Wiener Zeitung | 1819–1824, 1828, 1830, 1832–1834, 1836–1843, 1845­–1899 |
|  |  |  |
| Local papers | Neues Wiener Tagblatt | 1867–1877, 1883­–1899 |
|  | Morgen-Post | 1854­–1886 |
|  | Grazer Volksblatt | 1868–1899 |
|  | Grazer Zeitung | 1851, 1858­–1866, 1869–1877 |
|  | Linzer Volksblatt | 1869–1875, 1877–1879, 1881–1899 |
|  | Linzer Tages-Post | 1865–1899 |
|  | Linzer Abendbote | 1855, 1856, 1864–1868 |
|  | Salzburger Chronik für Stadt und Land | 1865–1889, 1891–1899 |
|  | Salzburger Volksblatt | 1871–1899 |
|  | Salzburger Zeitung | 1853–1877 |
|  | Innsbrucker Nachrichten | 1854–1899 |
|  | Klagenfurter Zeitung | 1819–1821, 1824, 1825, 1835, 1836, 1838, 1841, 1842, 1846–1849, 1853, 1854, 1856–1875 |
|  |  |  |
| Emerging Mass Press | Illustrirte Wiener Extrablatt | 1872–1875 |
|  | Neues Wiener Journal | 1893–1899 |
|  | Wiener Allgemeine Zeitung | 1880–1889 |
|  |  |  |
| Present-day newspapers | Kronen Zeitung | 2018–2020 |
|  | Heute | 2018–2020 |
|  | Kleine Zeitung | 2018–2020 |
|  | Kurier | 2018–2020 |
|  | Der Standard | 2018–2020 |
|  | OÖ Nachrichten | 2018–2020 |
|  | Die Presse | 2018–2020 |
|  | Tiroler Tageszeitung | 2018–2020 |
|  | Salzburger Nachrichten | 2018–2020 |
|  | Vorarlberger Nachrichten | 2018–2020 |
|  |  |  |

*Note.* Newspapers were selected based on a high circulation in the nineteenth century in the geographic region of the present state of Austria, their representation of different parts of the monarchy, different newspaper types, and their availability in the ANNO database. Of note, most of the newspapers were not available throughout the entire nineteenth century; some papers dis-, then re-appeared, some papers were published, but not available in ANNO. Importantly, multiple newspapers did not produce any search results for suicide reports in certain years. These years have been omitted in this list to display merely the years which yielded analyzable articles.

| **OSM Table 2**  *Results of ARIMA Modeling for the Test of Hypothesis 2: Predicting Suicide Rates by the Quantity of Low-Quality Reporting and Controls* | | | | | |
| --- | --- | --- | --- | --- | --- |
| *Variables* |  | *B* | *SE* | *z* | *p* |
| *Controls*  Economy (Consumer Price Index)  Politics (Democratization Index)  Freedom of Academic and Cultural Expression  Freedom of Religion  Women’s Civil Liberties Index  Rule of Law Index  *Conceptual Variable*  Quantity of Low-Quality Reporting |  | 0.00  0.35  0.09  -4.57  8.32  -19.78  7.40 | 0.00  1.29  0.44  2.00  7.51  17.52  2.77 | 0.18  0.27  0.21  -2.29  1.11  -1.13  2.68 | .855  .784  .836  .022  .268  .259  .007 |
| *Note.* The *auto.arima* function identified an ARIMA (0,1,3) model. This ARIMA model accounted for the autocorrelation in the time series as indicated by the Ljung-Box Q statistic on residual autocorrelation that was not significant (*p* = .90). Moving average terms are not reported in this table. | | | | | |

**OSM Table 3**

*Number of Available Articles and Descriptive Statistics of the Four Target Quality*

*Concepts Over Time*

|  |  |  | *Method* | |  | *Location* | |  | *Identification-evoking potential* | |  | *Prominence* | |
| --- | --- | --- | --- | --- | --- | --- | --- | --- | --- | --- | --- | --- | --- |
| Year | *n* |  | *M* | *SD* |  | *M* | *SD* |  | *M* | *SD* |  | *M* | *SD* |
| 1819 | 3 |  | 1.33 | 1.15 |  | 1.00 | 0.00 |  | 1.67 | 0.58 |  | 0.00 | 0.00 |
| 1820 | 5 |  | 1.00 | 1.00 |  | 1.00 | 0.71 |  | 2.40 | 1.52 |  | 0.00 | 0.00 |
| 1821 | 5 |  | 0.40 | 0.89 |  | 0.40 | 0.55 |  | 1.40 | 1.67 |  | 0.20 | 0.45 |
| 1822 | 4 |  | 1.00 | 0.00 |  | 0.50 | 0.58 |  | 1.50 | 1.29 |  | 0.75 | 0.50 |
| 1823 | 2 |  | 0.50 | 0.71 |  | 1.50 | 0.71 |  | 2.00 | 2.83 |  | 0.00 | 0.00 |
| 1824 | 4 |  | 0.50 | 1.00 |  | 1.25 | 0.50 |  | 0.75 | 1.50 |  | 0.25 | 0.50 |
| 1825 | 1 |  | 2.00 |  |  | 2.00 |  |  | 1.00 |  |  | 0.00 |  |
| 1826 | 0 |  |  |  |  |  |  |  |  |  |  |  |  |
| 1827 | 0 |  |  |  |  |  |  |  |  |  |  |  |  |
| 1828 | 2 |  | 1.00 | 1.41 |  | 1.00 | 0.00 |  | 1.00 | 1.41 |  | 0.50 | 0.71 |
| 1829 | 0 |  |  |  |  |  |  |  |  |  |  |  |  |
| 1830 | 2 |  | 0.50 | 0.71 |  | 1.00 | 0.00 |  | 1.00 | 1.41 |  | 0.50 | 0.71 |
| 1831 | 0 |  |  |  |  |  |  |  |  |  |  |  |  |
| 1832 | 1 |  | 0.00 |  |  | 2.00 |  |  | 3.00 |  |  | 1.00 |  |
| 1833 | 1 |  | 2.00 |  |  | 2.00 |  |  | 4.00 |  |  | 2.00 |  |
| 1834 | 3 |  | 0.67 | 1.15 |  | 0.00 | 0.00 |  | 1.00 | 1.73 |  | 0.00 | 0.00 |
| 1835 | 1 |  | 0.00 |  |  | 0.00 |  |  | 4.00 |  |  | 0.00 |  |
| 1836 | 12 |  | 0.42 | 0.67 |  | 0.83 | 0.58 |  | 1.50 | 1.24 |  | 0.08 | 0.29 |
| 1837 | 7 |  | 0.57 | 0.98 |  | 1.00 | 0.58 |  | 1.57 | 1.27 |  | 0.14 | 0.38 |
| 1838 | 6 |  | 0.00 | 0.00 |  | 1.00 | 0.00 |  | 0.33 | 0.82 |  | 0.17 | 0.41 |
| 1839 | 4 |  | 0.00 | 0.00 |  | 1.00 | 0.00 |  | 1.00 | 1.15 |  | 0.00 | 0.00 |
| 1840 | 2 |  | 0.50 | 0.71 |  | 1.00 | 0.00 |  | 0.00 | 0.00 |  | 0.50 | 0.71 |
| 1841 | 10 |  | 0.70 | 0.95 |  | 0.90 | 0.74 |  | 1.80 | 1.55 |  | 0.50 | 0.53 |
| 1842 | 9 |  | 0.89 | 1.05 |  | 0.67 | 0.71 |  | 2.00 | 2.00 |  | 0.22 | 0.44 |
| 1843 | 4 |  | 1.50 | 0.58 |  | 1.25 | 0.50 |  | 1.75 | 2.06 |  | 1.00 | 0.82 |
| 1844 | 0 |  |  |  |  |  |  |  |  |  |  |  |  |
| 1845 | 7 |  | 0.71 | 0.95 |  | 1.43 | 0.53 |  | 2.71 | 1.25 |  | 0.14 | 0.38 |
| 1846 | 8 |  | 0.50 | 0.76 |  | 0.63 | 0.52 |  | 2.00 | 1.20 |  | 0.00 | 0.00 |
| 1847 | 6 |  | 1.33 | 1.03 |  | 1.00 | 0.89 |  | 2.17 | 1.33 |  | 0.17 | 0.41 |
| 1848 | 17 |  | 1.18 | 0.95 |  | 0.71 | 0.59 |  | 1.47 | 1.12 |  | 0.00 | 0.00 |
| 1849 | 24 |  | 1.29 | 0.86 |  | 1.25 | 0.74 |  | 2.54 | 1.64 |  | 0.04 | 0.20 |
| 1850 | 32 |  | 0.88 | 0.91 |  | 1.03 | 0.69 |  | 1.72 | 1.53 |  | 0.00 | 0.00 |
| 1851 | 41 |  | 1.32 | 0.85 |  | 1.17 | 0.54 |  | 2.29 | 1.10 |  | 0.00 | 0.00 |
| 1852 | 56 |  | 1.21 | 0.87 |  | 1.04 | 0.50 |  | 2.04 | 1.09 |  | 0.00 | 0.00 |
| 1853 | 72 |  | 1.50 | 0.73 |  | 1.29 | 0.68 |  | 2.15 | 1.10 |  | 0.04 | 0.20 |
| 1854 | 98 |  | 1.34 | 0.77 |  | 1.33 | 0.61 |  | 1.97 | 0.96 |  | 0.01 | 0.10 |
| 1855 | 99 |  | 1.51 | 0.69 |  | 1.21 | 0.63 |  | 1.91 | 1.03 |  | 0.51 | 0.50 |
| 1856 | 144 |  | 1.45 | 0.80 |  | 1.41 | 0.68 |  | 2.29 | 1.21 |  | 0.40 | 0.51 |
| 1857 | 139 |  | 1.24 | 0.88 |  | 1.19 | 0.67 |  | 2.12 | 1.19 |  | 0.31 | 0.46 |
| 1858 | 130 |  | 1.33 | 0.83 |  | 1.33 | 0.68 |  | 2.17 | 1.15 |  | 0.33 | 0.47 |
| 1859 | 109 |  | 1.54 | 0.75 |  | 1.45 | 0.75 |  | 2.25 | 1.03 |  | 0.31 | 0.47 |
| 1860 | 129 |  | 1.33 | 0.89 |  | 1.23 | 0.69 |  | 2.69 | 1.37 |  | 0.56 | 0.51 |
| 1861 | 180 |  | 1.44 | 0.81 |  | 1.24 | 0.78 |  | 2.68 | 1.33 |  | 0.43 | 0.51 |
| 1862 | 187 |  | 1.43 | 0.77 |  | 1.38 | 0.73 |  | 2.49 | 1.20 |  | 0.44 | 0.51 |
| 1863 | 180 |  | 1.56 | 0.73 |  | 1.16 | 0.72 |  | 2.80 | 1.23 |  | 0.37 | 0.48 |
| 1864 | 246 |  | 1.49 | 0.78 |  | 1.22 | 0.69 |  | 2.52 | 1.19 |  | 0.50 | 0.52 |
| 1865 | 297 |  | 1.49 | 0.74 |  | 1.38 | 0.64 |  | 2.74 | 1.32 |  | 0.56 | 0.51 |
| 1866 | 273 |  | 1.52 | 0.75 |  | 1.45 | 0.71 |  | 2.83 | 1.35 |  | 0.52 | 0.51 |
| 1867 | 296 |  | 1.63 | 0.70 |  | 1.45 | 0.75 |  | 2.55 | 1.22 |  | 0.62 | 0.49 |
| 1868 | 332 |  | 1.53 | 0.75 |  | 1.39 | 0.74 |  | 2.72 | 1.34 |  | 0.54 | 0.50 |
| 1869 | 313 |  | 1.45 | 0.78 |  | 1.42 | 0.82 |  | 2.90 | 1.32 |  | 0.58 | 0.49 |
| 1870 | 312 |  | 1.59 | 0.76 |  | 1.47 | 0.79 |  | 3.10 | 1.37 |  | 0.65 | 0.48 |
| 1871 | 380 |  | 1.50 | 0.76 |  | 1.39 | 0.83 |  | 3.06 | 1.38 |  | 0.66 | 0.48 |
| 1872 | 410 |  | 1.55 | 0.74 |  | 1.35 | 0.82 |  | 2.92 | 1.41 |  | 0.71 | 0.49 |
| 1873 | 441 |  | 1.57 | 0.74 |  | 1.53 | 0.86 |  | 3.20 | 1.40 |  | 0.71 | 0.50 |
| 1874 | 476 |  | 1.52 | 0.75 |  | 1.46 | 0.81 |  | 2.96 | 1.44 |  | 0.76 | 0.44 |
| 1875 | 473 |  | 1.58 | 0.73 |  | 1.51 | 0.84 |  | 3.14 | 1.38 |  | 0.75 | 0.46 |
| 1876 | 377 |  | 1.58 | 0.72 |  | 1.47 | 0.81 |  | 3.14 | 1.44 |  | 0.69 | 0.48 |
| 1877 | 405 |  | 1.53 | 0.76 |  | 1.47 | 0.85 |  | 3.20 | 1.48 |  | 0.71 | 0.48 |
| 1878 | 305 |  | 1.56 | 0.75 |  | 1.47 | 0.83 |  | 3.15 | 1.54 |  | 0.58 | 0.55 |
| 1879 | 306 |  | 1.54 | 0.76 |  | 1.40 | 0.84 |  | 3.25 | 1.47 |  | 0.63 | 0.48 |
| 1880 | 330 |  | 1.52 | 0.82 |  | 1.52 | 0.82 |  | 3.30 | 1.42 |  | 0.71 | 0.48 |
| 1881 | 344 |  | 1.56 | 0.73 |  | 1.50 | 0.89 |  | 3.56 | 1.40 |  | 0.80 | 0.43 |
| 1882 | 358 |  | 1.50 | 0.78 |  | 1.51 | 0.82 |  | 3.60 | 1.35 |  | 0.77 | 0.43 |
| 1883 | 390 |  | 1.39 | 0.87 |  | 1.53 | 0.83 |  | 3.50 | 1.47 |  | 0.75 | 0.46 |
| 1884 | 390 |  | 1.53 | 0.78 |  | 1.53 | 0.86 |  | 3.55 | 1.33 |  | 0.78 | 0.42 |
| 1885 | 390 |  | 1.58 | 0.72 |  | 1.54 | 0.86 |  | 3.45 | 1.40 |  | 0.80 | 0.41 |
| 1886 | 390 |  | 1.56 | 0.79 |  | 1.56 | 0.89 |  | 3.63 | 1.39 |  | 0.73 | 0.45 |
| 1887 | 360 |  | 1.49 | 0.79 |  | 1.49 | 0.89 |  | 3.64 | 1.33 |  | 0.75 | 0.44 |
| 1888 | 363 |  | 1.48 | 0.73 |  | 1.60 | 0.85 |  | 3.65 | 1.29 |  | 0.79 | 0.41 |
| 1889 | 360 |  | 1.49 | 0.76 |  | 1.57 | 0.91 |  | 3.73 | 1.35 |  | 0.82 | 0.39 |
| 1890 | 302 |  | 1.43 | 0.76 |  | 1.58 | 0.84 |  | 3.46 | 1.46 |  | 0.79 | 0.42 |
| 1891 | 340 |  | 1.59 | 0.76 |  | 1.51 | 0.79 |  | 3.64 | 1.32 |  | 0.74 | 0.44 |
| 1892 | 335 |  | 1.50 | 0.76 |  | 1.46 | 0.82 |  | 3.59 | 1.39 |  | 0.75 | 0.44 |
| 1893 | 372 |  | 1.45 | 0.78 |  | 1.41 | 0.84 |  | 3.57 | 1.27 |  | 0.70 | 0.47 |
| 1894 | 369 |  | 1.44 | 0.80 |  | 1.49 | 0.88 |  | 3.60 | 1.40 |  | 0.75 | 0.45 |
| 1895 | 390 |  | 1.48 | 0.79 |  | 1.64 | 0.87 |  | 3.89 | 1.22 |  | 0.71 | 0.46 |
| 1896 | 388 |  | 1.55 | 0.74 |  | 1.60 | 0.95 |  | 3.87 | 1.21 |  | 0.76 | 0.43 |
| 1897 | 361 |  | 1.58 | 0.71 |  | 1.67 | 0.81 |  | 3.76 | 1.18 |  | 0.78 | 0.42 |
| 1898 | 360 |  | 1.49 | 0.79 |  | 1.51 | 0.89 |  | 3.74 | 1.23 |  | 0.76 | 0.43 |
| 1899 | 358 |  | 1.53 | 0.75 |  | 1.55 | 0.83 |  | 3.77 | 1.19 |  | 0.72 | 0.45 |
|  |  |  |  |  |  |  |  |  |  |  |  |  |  |
